# Editorial: Integrated Omics for Defining Interactomes

**DOI:** 10.3389/fphys.2020.00081

**Published:** 2020-02-14

**Authors:** Sudipto Saha, Rob M. Ewing

**Affiliations:** ^1^Division of Bioinformatics, Bose Institute, Kolkata, India; ^2^Division of Computational and Systems Biology, School of Biological Sciences, University of Southampton, Southampton, United Kingdom

**Keywords:** multi-omics, protein–protein interactions, integrative analysis, heterogeneous network, computational tools

Integration of multiple types of omics data is a powerful strategy to aid the understanding of the combined influence of complex biological processes at the cellular level. The focus on the application of multiple omics techniques to the same biological question is rapidly increasing due to the advent of sophisticated and robust instrumentation, such as Next-Generation Sequencing and mass spectrometry. In addition, the application of statistical tools and new computational approaches, such as iOmicsPASS, allow for the integration, analysis, and visualization of the integrated multi-omics data (Koh et al., 2019). Seminal large-scale multi-omics studies, such as The Cancer Genome Atlas (TCGA), have paved the way for the application of integrated multi-omics to other systems, including host-pathogen interactions and the analysis of the pluripotency regulatory network in stem cells, but still there is a need to explore this approach to reveal the complex networks in molecular systems biology (Chakravorty et al., 2018). The cross-talk between multi-omics layers, including transcriptomics, proteomics, and metabolomics, in several biological systems will ultimately aid in understanding the complexity of heterogeneous interactomes.

In this Research Topic, contributors have addressed the application of multi-omics to diverse biological problems as well as the methodological challenges. Viral-host protein–protein interactions have been studied using several experimental methods, including Yeast Two-Hybrid (Y2H) and Affinity Purification-Mass Spectrometry (AP-MS), followed by comprehensive description of computational tools for the filtering of false positives and ranking. The experimental methods were broadly classified into two classes: *ex situ*, which includes assays that occur outside the normal physiological conditions [Y2H, GST pull down, and Nucleic Acid-Programmable Protein Assay (NAPPA)] and *in situ*, which includes assays that occur inside host cells e.g., AP-MS and proximity-dependent labeling. Though the *in situ* assays are more commonly used in studying viral–host interactions, they do require the expertise for the analysis of mass-spectrometry data. The advantages and disadvantages of three (ComPASS, SAINT, and MiST) computational tools and scoring metrics for AP-MS data were described and compared. The advantage of using the MiST approach, whereby the prey protein abundance, reproducibility, and prey specificity are used, were discussed in contrast to the other methods. Interpretation of the viral-host protein-protein interactions may be performed using Viruses.STRING (Cook et al., 2018) and the Gene Ontology. Researchers should, however, be cautious in interpreting the results; related viruses may undergo mutations and thus may not be captured in the same interactome when comparing between different strains of same viral species.

In understanding the disease biology, there are relatively few direct studies of molecular interactions (e.g., protein–protein interactions) as compared to high numbers of genome-scale quantitative omics studies using transcriptomics, proteomics, and metabolomics. These multi-omics studies allow researchers to explore the functional or indirect interactions between molecules. Hawe et al. describe the statistical basis for inferring indirect interactions from multi-omics data using conditional dependencies (partial correlations) networks, a graphical lasso, and Bayesian models. The authors highlighted the mixed graphical models (MGM) for building heterogeneous networks from multi-omics data. The authors also mentioned GENIE3 (for Gene Network Inference with Ensemble of trees), a tree-based method used for constructing homogeneous networks from gene expression data, and it can also be applied to heterogeneous network construction. Multi-omics data integration for heterogeneous interactomes can be done step by step using GWAS-SNPs association with mRNA expression (eQTLs), protein abundance (pQTL), and DNA methylations (meQTLs). At present, several strategies are available for integration of different molecular data, and inference efforts from these integrated networks may still be improved.

miRNA-seq studies play an important role in the mechanistic understanding of post-transcriptional control and identifying regulatory miRNA-Gene-TF networks. Wang et al. performed an integrative analysis of miRNA, mRNA, and DNA methylation in exploring the role of transforming growth factor-beta (TGF-β1) on kidney glomerular mesangial cells. The authors identified 5,140 significantly differentially expressed (DE) genes in TGF-β1-treated cells, whereas an integrative analysis of miRNA target genes and miRNA was reduced to 122 DE-mRNAs and 11DE-miRNAs. In addition, an integrative analysis of DNA-methylated genes and DE-mRNA gene sets were used for a pathway analysis that revealed five major pathways, including epithelial adherens junction signaling. Their findings using integrative analyses reduced the list of predictive gene targets. Denkiewicz et al., described a method for integrating the miRNA-seq of breast invasive carcinoma (BRCA) and survival analysis of 231 patients' clinical data obtained from The Cancer Genome Atlas (TCGA). The authors identified the top 100 miRNAs and grouped them in to two classes: four-star miRNAs that are involved in all four subtypes of breast cancer (Luminal A, Luminal B, HER2-Enriched, and Basal-Like) and one-star miRNAs that are present only in a specific subtypes. Machine learning tools achieved an average accuracy of 95.10% in classifying breast cancer sub-types using a four-star miRNAs dataset. Furthermore, the authors reported a combined network of miRNA-Gene-TF, where several important transcriptional factors were present, such as MYC, ESR1, BRCA1, and HIF1A. Stumpf and MacArthur describe a novel principal component analysis-type approach to predict regulatory network patterns from the time-course single cell protein expression data of mouse embryonic stem cells. Three distinct regulatory states for naïve, formative, and early primitive endoderm were identified.

Integrated biological networks based on multi-omics data help in understanding the pathogenesis of complex diseases. Sumathipala et al. describe a method to predict lncRNA-disease associations using a random walk network diffusion algorithm and tripartite network consisting of lncRNA–protein, protein–protein, and protein–disease associations. The proposed method named as lncRNA ranking by NetwOrk DiffusioN (LION) was evaluated for prediction of cardiovascular diseases and cancer and neurological diseases by using experimentally verified lncRNAs, and it was observed that this method achieved an overall AUC values of greater than 90%. LION also performed well in predicting LncRNAs for breast, blood, ovarian, and bladder cancer. Several of the top 50 predicted lncRNAs using LION for these specific cancers were found to be experimentally validated. A main limitation is, however, the bias inherent in the existing datasets of lncRNA-proteins, protein-protein interactions and protein-disease associations initially used to develop the algorithm. Choi et al. show how circulating blood miRNAs and proteins can be used in network-based integrative analysis in studying eight obese insulin-resistant (OIR) and nine lean insulin-sensitive (LIS) individuals. The authors generated the expression data of miRNAs (MiRXES) and blood plasma proteins (LC-MS/MS) and reported 374 differentially expressed circulating miRNAs and 40 plasma proteins, which were further linked using TargetScan. The authors reported predictive subnetworks by merging these expression datasets with two biological networks (TargetScan map and protein–protein interactions) using iOmicsPASS (Koh et al., 2019) and finally identified several miRNA-protein pairs with same tissue of origin, like adipose and liver tissue. This study is unique since it focused on the profiled plasma secretome of OIR and LIS subjects; however, the main limitation is the small sample size.

In summary, in this Research Topic contributors have provided examples of both the growing number of applications of integrated multi-omics analyses to diverse biological systems as well as the rapid methodological innovations that have been made to develop tools and approaches for integrating the resulting data.

## Author Contributions

Both authors listed have made a substantial, direct and intellectual contribution to the work, and approved it for publication.

### Conflict of Interest

The authors declare that the research was conducted in the absence of any commercial or financial relationships that could be construed as a potential conflict of interest.
